# NEXT STEPs: Evidence-Based Recruitment and Retention for Nursing Home Clinical Trials

**DOI:** 10.1093/geroni/igaf122.452

**Published:** 2025-12-31

**Authors:** Sheryl Zimmerman, Johanna Hickey, Lea Efird-Green, Jennifer Carnahan, Gail Towsley, Alice Bonner, Jasmine Altizer

**Affiliations:** University of North Carolina at Chapel Hill, Chapel Hill, North Carolina, United States; The University of North Carolina at Chapel Hill, Chapel Hill, North Carolina, United States; The University of North Carolina at Chapel Hill, Chapel Hill, North Carolina, United States; Indiana University Center for Aging Research, Indianapolis, Indiana, United States; University of Utah, Salt Lake City, Utah, United States; Institute for Healthcare Improvement, Boston, Massachusetts, United States; New York University, New York, New York, United States

## Abstract

The National Institute on Aging funded NEXT STEPs (Nursing home EXplanatory clinical Trials: Supporting Transformation by Enhancing Partnerships) to promote the successful conduct of nursing home clinical trials. Three of NEXT STEPs’ key elements recognize the importance of the relationship between researchers and those participating in research: recruiting nursing homes and participants; cultivating engagement throughout the process; and following up and following through on communication to share results and promote sustainable implementation. NEXT STEPs’ Recruitment and Retention Core focuses on these areas, a key effort being the development of a range of evidence-based best practice resources for use by those who conduct research and participate in research. To begin this effort, Core members searched the literature and conferred with experts to develop an extensive and evolving list of best practice topics – such as strategies to help researchers convey the importance of research, and participants to understand their rights as a research partner – and determine the optimal format to convey the best practices (e.g., checklists, short videos). The first document created for researchers is entitled “Words Matter,” employing a “use this/not that” format (e.g., use “choose not to” rather than “noncompliant”). Feedback on materials are obtained from diverse partner workgroups before they are finalized, and their utility is evaluated as they are used in NEXT STEPs’ efforts. This session will overview the topics identified important for recruitment and retention, the process of topic selection and material development, and materials developed to date; audience feedback and recommendations will be solicited.

